# Structural perturbations of substrate binding and oxidation state changes in a lytic polysaccharide monooxygenase

**DOI:** 10.1007/s00775-022-01966-z

**Published:** 2022-10-08

**Authors:** Paul H. Walton, Gideon J. Davies

**Affiliations:** Department of Chemistry, University of York, Heslington, York, YO10 5DD UK

**Keywords:** LPMO, Copper, Errors, Protein structure

## Abstract

LPMOs are enzymes which catalyse the oxidation of a C-H bond within polysaccharides, leading to their oxidative cleavage. To achieve this, LPMOs employ highly reactive oxidising intermediates, the generation of which is likely coupled to substrate binding to the enzyme. The nature of this coupling is unknown. Here we report a statistical comparison for four three-dimensional structures of an AA9 LPMO crystallised in the same space group but in different oxidation and substrate-binding states, to determine which significant structural perturbations occur at the enzyme upon either oxidation state change or the binding of substrate. In a novel step, we determine the global random error associated with the positional coordinates of atoms using the method of moments to ascertain the statistical estimators of Gaussian distributions of pairwise RMS differences between individual atoms in different structures. The results show that a change in the oxidation state of the copper leads to no significant structural changes, and that binding of the substrate leads to a single change in the conformation of a tryptophan residue. This tryptophan has previously been identified as part of a charge transfer pathway between the active site and the external surface of the protein, and the structural change identified herein may be part of the substrate-enzyme coupling mechanism.

**Graphical abstract:**

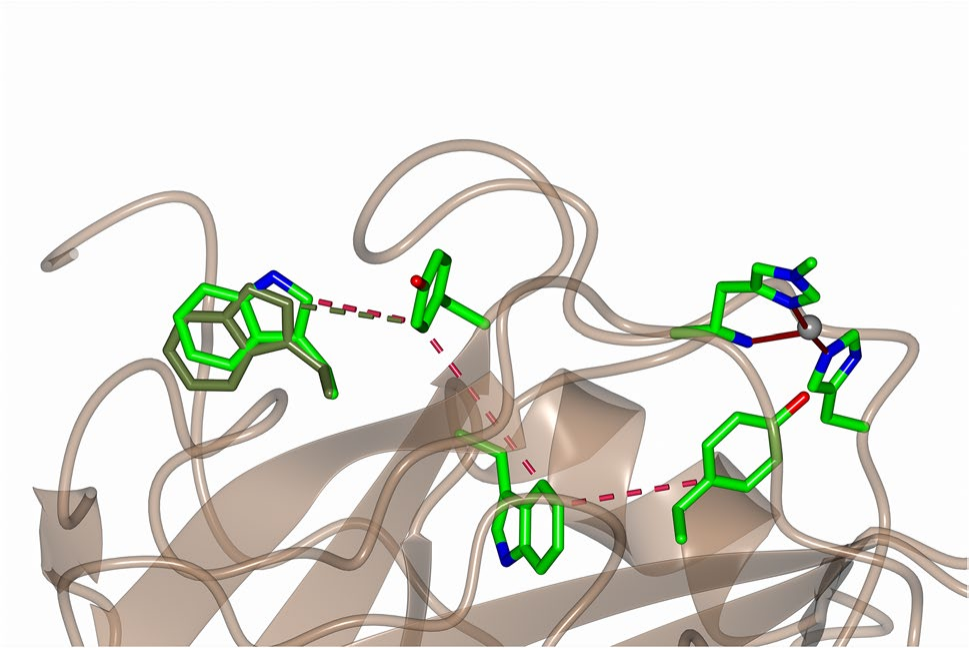

**Supplementary Information:**

The online version contains supplementary material available at 10.1007/s00775-022-01966-z.

## Introduction

**Fig. 1:**
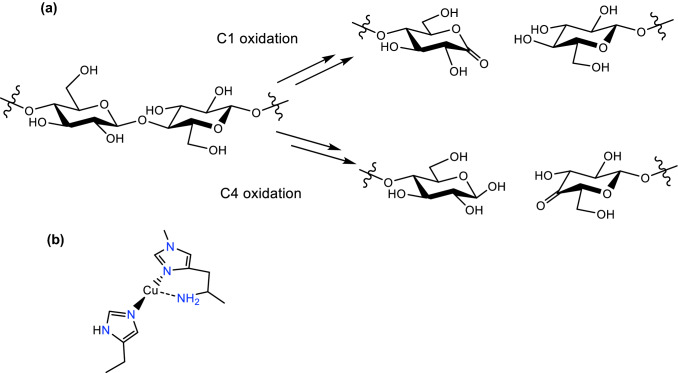
**a** Substrate oxidation catalysed by LPMOs, and **b** histidine brace active site of an AA9 LPMO in its Cu(I) oxidation state

Lytic polysaccharide monooxygenases (LPMOs, sometimes known as PMOs) are copper-containing enzymes that catalyse the oxidative cleavage of polysaccharides by oxygen and/or hydrogen peroxide [1–3]. LPMOs catalyse the oxidative cleavage of polysaccharides using O_2_/reducing agent or H_2_O_2_ as cofactors [4–6]. Interest in LPMOs has increased recently because of the use of these enzymes in the production of second-generation bioethanol and also for their unusual active site, in which a single copper ion is coordinated by a histidine brace (Fig. 1) [7]. LPMOs are delineated into eight distinct genomic classes, AA9-AA10, AA12-AA17, some of which feature an unusual methylation of the N-terminal histidine that forms part of the histidine brace [8–10].

There has also been interest in the mechanism of action of LPMOs, with attention focussed on the means by which the enzymes generate highly oxidising intermediates and also how these enzymes are able to avoid auto-oxidation of the protein backbone [11, 12]. In this latter aspect, the use of H_2_O_2_ as a cofactor can lead to substantial protein damage suggesting that peroxide acts as a shunt and that LPMOs use O_2_ as their natural substrate and, furthermore, that O_2_ activation is coupled to substrate binding, such that generation of highly oxidising intermediates only occurs in the presence of substrate. Indeed, spectroscopic studies have already demonstrated that the active site copper undergoes changes to the energetics of its d-orbital manifold upon substrate binding, the mechanism of which is associated with the formation of a network of hydrogen bonds between substrate and active site [13]. Wider structural changes in the protein, however, have not yet been considered. It is in this context that we present here a statistical analysis of the structural changes which occur to an LPMO on both oxidation state change and also on substrate binding. Our findings are that a change in oxidation state at the copper is not associated with any significant movement of amino acids, whereas binding of an oligosaccharide is associated with a conformational change in the side chain of a single tryptophan residue. This residue has previously been identified in this particular LPMO as being part of an electron transfer chain between the copper active site and the external surface of the protein [14]. As such, substrate binding may be associated with the gating of electron transfer in the mechanism of action of the enzyme.

## Methods

The crystal structure coordinates and associated displacement parameters of an AA9 LPMO from *Lentinus similis* (LsAA9) were taken from the PDB database (5ACH, 5ACG, 5ACF, 5ACJ) [15]. This collection of four structures contains those of an LPMO in which a substrate (cellotriose in 5ACF and 5ACJ) is bound to the active site. The structures of this LPMO have also been determined in the absence of substrate (5ACH, 5ACG) and in its two different Cu oxidation states (Cu(II) 5ACG, 5ACF and Cu(I) 5ACH 5ACJ), thus providing a set of four structures in which both substrate and Cu oxidation state are varied. The structures are largely isostructural (save the presence of substrate), crystallise in the same space group (P4_1_32), were all refined with similar restraints, and the crystals were prepared at the same pH under very similar conditions. In many ways, therefore, this set of structures is ideal for direct comparison and thus evaluation of the structural differences brought about by substrate binding and/or oxidation state change. There were, however, differences in the data collection strategies, in which the structures with Cu(II) oxidation states (5ACG, 5ACF) were collected at low doses of X-rays to minimise photo-reduction of the metal ion (20–30% beam intensity). Accordingly, the data collection statistics for these two structures are poorer than the full X-ray exposure structures (5ACH, 5ACJ). Also, one structure (5ACH) was refined with anisotropic displacement parameters. These differences in collection/refinement strategies necessitate that any comparison of structures takes into account the random errors associated with the relative positions of atoms in the structures, such that any claims of meaningful differences between structures are statistically significant.

Any comparative study of crystal structures contains many potential pitfalls. Multiple experimental factors contribute to the apparent positional differences between equivalent atoms in different structures, some of which (e.g. refinement re/constraints, correlation between displacement factors and atomic coordinates) can artificially lower the apparent random error in difference. As such caution is required in drawing any functional conclusion about the differences in atomic positions between structures, without first performing an analysis of the sources and sizes of errors in the positional coordinates of atoms. This caution has not necessarily been evident in previous comparisons of LPMO structures.

In the cases in which a full matrix least squares refinement has been carried out in the solution of the structures, then the estimates of the precision of atomic positions are determined from the full variance–covariance matrix. These esds can then be used in multivariate comparisons of all or part of the structures from which measures of statistical significance can be obtained. Since, however, refinement of crystal structures is typically carried out with blocked least squares matrices, the full variance–covariance matrix is unavailable. Therefore, other methods are employed to determine the degree of global random error in atomic coordinates within the majority of protein structures. For instance, the widely used Luzatti method estimates an upper value for the global random error by examining differences in calculated and observed structure factors within shells of sin*θ*/*λ*. Another method by Cruikshank et al. calculates a diffraction precision indicator (DPI) which is an index of random error [16].

DPI = 0.7(*N*/*P*)^1/2^ *C*^−1/3^ *d*_min_ *R*

*N* = number of refined atoms, *P* = degrees of freedom, *C* = completeness, *d*_min_ = resolution, *R* = crystallographic R factor. The DPI can be easily calculated from published collection parameters, and, moreover, has been shown to be correlated to the esds of carbon atoms in small molecules. An estimated error of the difference in positions of two atoms (σ_1,2_) can, therefore, be calculated from the esds of the individual atoms (σ_*n*_) using the equation:

σ_1,2_ = {3(σ_1_^2^ + σ_2_^2^)}^0.5^

Displacement factor-corrected RMS differences between atoms can then be compared to the estimated error from the DPI and assessed for their significance.

Notwithstanding however, the convenience of the DPI and associated methods of error estimation, these approaches only estimate the global random error in the positional parameters of atoms, which is likely to be a significant underestimate in the positions of atoms which have high thermal parameters. In this regard, several previous studies of error estimates have empirically equated the random error in individual atomic positional parameters with the displacement factor of that atom. These methods assume a functional correlation between the displacement parameter and the error in the individual atomic positions.

However, as pointed out by other authors, this method, while appealing from a utility point of view, is vulnerable to artefacts arising from differences in refinement methods and other sources of systematic error. Somewhat in light of this concern, other methods have sought to estimate a random error of positional parameters by using the displacement-parameter-weighted RMS differences between pairs of equivalent atoms in two different structures of the same protein [17, 18]. For a given range of displacement parameters, these pairwise differences then form a distribution, from which an associated mean and variance can be calculated. The mean of the distribution can then be used as an estimate of the random error associated with the positional parameters of the atom. Moreover, the variance of the distribution can also be used when evaluating the significance of any difference in the positional parameters. Indeed, these methods work well for closely related structures collected/refined using similar approaches and where the structural perturbations are small, since they directly determine a pairwise error of comparison of the position parameters of atoms in the two structures, and do not rely on estimates of error from indicators such as displacement parameters and collection statistics.

It is in this context that we describe herein a method which is tailored to determining the significance, if any, of differences in positional parameters between equivalent atoms in closely-related but different structures of LPMOs. Our aim is to ascertain the effects of a change in the oxidation state of the copper ion, and the perturbations brought about by the binding of substrate. Our approach is analogous to that of Peters-Libeu et al. in which pairwise RMS differences in the positional parameters of equivalent atoms between related structures are used to estimate the random error of comparison between the two structures [18]. However, to mitigate the effects of correlation between displacement and positional parameters, rather than weighting the RMS differences by the average displacement parameters of a pair of equivalent atoms, we have adapted the approach of Stroud and Fauman to construct distributions of RMS differences within ‘windows’ of average displacement parameter values, such that potential artefacts arising from correlation do not lead to an underestimation of error for atoms with high displacement parameters [19].

Then, in contrast to Peters–Libeu et al. but in line with Stroud and Fauman, we have converted the resulting Maxwellian distributions (in accord with the scalar nature of the non-dependent variable), to Gaussian coordinate distributions. The resulting Gaussian distributions form the basis of the random error associated with the positional parameters of individual atoms as a function of their displacement parameter. Notably, in an extension to previous studies, we have calculated estimators of the Gaussian distributions using the method of moments (MoM). The advantage of the MoM is that it proceeds under weak assumptions about the underlying distribution and, importantly, affords consistent estimators when the distributions are built from large datasets as is found in the comparison of protein structures. Moreover, the MoM is computationally light, allowing the determination of estimators for a large number of different B-factor ranges, as is required by our method described below.

## Construction of displacement-parameter-selected RMS difference distributions

**Fig. 2:**
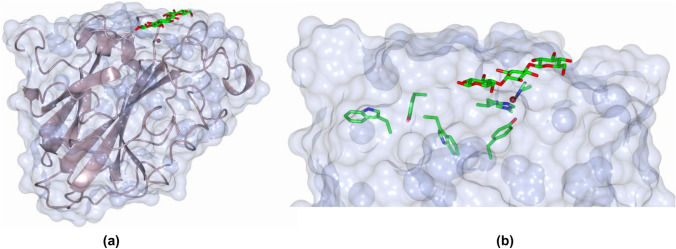
Representations of structures of LsAA9 LPMOs, **a** overall protein structure (bronze-coloured ribbons with a pale blue surface) with G3 substrate (represented as green cylinders), **b** detail of protein structure with amino acid aside chains involved in charge-transfer shown as green cylinders [15]

**Fig. 3:**
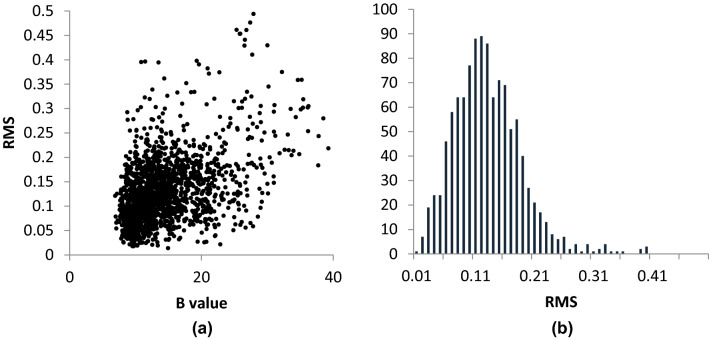
**a** Example of the scatter plot of average isotropic displacement parameters and RMS positional difference between pairs of equivalent atoms in two protein structures, and **b** histogram of RMS difference values constructed from a ‘window’ of displacement parameters (15 ± 6 Å^2^) of scatter plot in Fig. 3a. Data were taken from the pairwise comparison of 5ACF and 5ACG. All other distributions are given in the Supplementary Information

In general, each LsAA9 structure consists of a central beta sandwich around which polypeptide of non-descript secondary structure is evident (Fig. 2a). The histidine brace active site is situated on the edge of the protein at the end of a beta sheet. In two cases, a cellotriose substrate binds at the active site, spanning the histidine brace. The general method for the construction of RMS differences between equivalent atoms in two different structures, as a function of the average of their isotropic displacement factors, is as follows. For any two structures to be compared, drawn from any pair of 5ACF, 5ACG, 5ACH, 5ACJ, the coordinates of water molecules and the oligosaccharide substrate were first removed from the coordinate sets. Using Wincoot, the global RMS difference between all remaining non-hydrogen atoms was minimised, such that any small systematic displacement differences in unit cell axes were removed [20]. These differences are listed in Table 1 and are all small, in accord with the similarity in protein structure and collection/refinement methods. The adjusted coordinates of the two structures were then used to calculate a scatter plot of the RMS difference between the positions of equivalent atoms in the two datasets, the form of which is very similar to those presented in other papers in which there is a broad correlation between RMS difference and average displacement parameter value (Fig. 3). From these data, a B value was selected in which the B values of the atoms of the amino acids to be assessed fell and a range set around it (usually ± 2–5 Å^2^ dependent on the overall distribution). This range of B values was selected as to encompass most of the data (> 90%). A histogram was then constructed using running bins of RMS differences (0.01 intervals) and the populations of each of the RMS difference bins. The histograms in all cases had Maxwellian forms (Fig. 3b and Supplementary Information Fig. 1), as expected from the use of the three-dimensional scalar RMS difference as the categorical variable.

**Table 1:** Examples of displacement parameter range used in pairwise comparisons

Structures	Overall average RMS difference (all data), average displacement parameter of pairwise atoms	Displacement parameter range included in example distributions	Percentage of all data included in distribution
Cu(I) and Cu(II)	0.13 Å, 15 Å^2^	9–21 Å^2^	80.0%
5ACH and 5ACG			
Cu(I) and Cu(I)-G3	0.13 Å, 20 Å^2^	15–25 Å^2^	64.1%
5ACH and 5ACJ			
Cu(II) and Cu(II)-G3	0.15 Å, 15 Å^2^	9–21 Å^2^	77.8%
5ACG and 5ACF			
Cu(I)-G3 and Cu(II)-G3	0.17 Å, 19 Å^2^	14–24 Å^2^	70.6%
5ACJ and 5ACF

Maxwellian distributions do not straightforwardly yield estimators of variance and cannot easily form a basis on which to make estimates of the random error of a distribution of values. Therefore, Gaussian distributions of one-dimensional positional variables were constructed from the Maxwellian distributions described above using the method of Stroud and Fauman, which replaces the individual RMS difference values with their corresponding one-dimensional positional difference distributions (Δ*x*) [19]. The resulting Gaussian distributions were then used as the basis for the estimation of the random error of any comparison between any single pair of structures.

## Distribution estimators

Previous analyses of the resulting Gaussian distributions from pairwise comparison of coordinates have relied on standard methods of determining the distribution estimators (mean, variance, skew, kurtosis). Each of these methods works from underlying assumptions about the distribution of values, and can sometimes be computationally expensive (e.g. maximum likelihood methods). These assumptions also have the potential to lead to inconsistent estimators, thus introducing a source of systematic error of analysis. To overcome this issue, we have used herein a ‘method of moments’ (MoM) analysis of the Gaussian distributions. The MoM analysis proceeds under weak assumptions of the underlying distribution, and, moreover, yields consistent, albeit potentially biased, estimators for large datasets, ideal for the current analysis with datasets of over 1000 points. Additionally, the method is straightforward and computationally inexpensive.

**Fig. 4:**
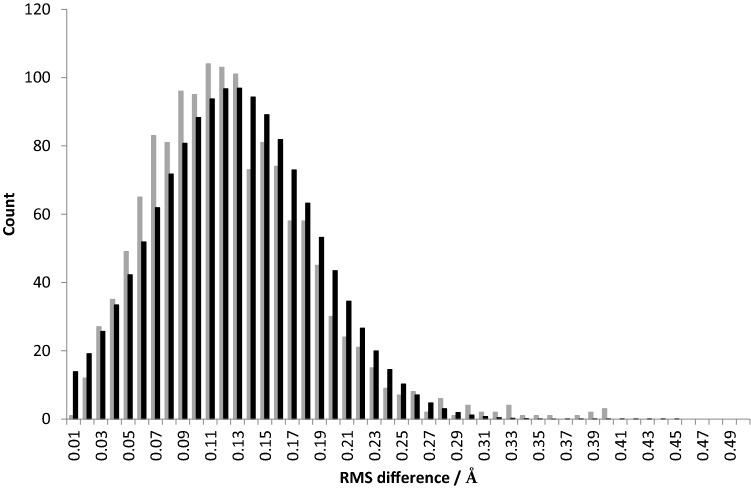
Histograms of RMS differences from the pairwise comparison of atomic coordinates (*B* values 6–21 Å^2^) in structures 5ACF and 5ACG: grey columns are experimental data, black columns are calculated from the method of moments values of ($\widehat{\mu }$) and ($\sqrt{\widehat{\sigma }}$) using the standard closed equation for a normal distribution

The MoM is described in standard statistical textbooks and is not repeated in detail here. Briefly, however, the following method was used. For each Gaussian distribution of the pairwise comparison of structures listed in Table 1, the population of pairwise RMS values was constructed for a total of *p* running bins at 0.01 intervals (Fig. 4, light grey columns). The fractional populations of each of these bins (X_i_) were calculated by dividing the number in each bin by the total number of RMS values. The individual moments for this distribution were then calculated using standard formulae, as follows.

$${M}_{1}=\sum_{i=0.01}^{0.01p}i{X}_{i}$$

$${M}_{n}=\sum_{i=0.01}^{0.01p}{{X}_{i}\left(i-{M}_{1}\right)}^{n}$$

The first moment, *M*_1_, is directly equated with the estimator for the mean of the distribution, $\widehat{\mu }$, and the second moment, *M*_2_, is equated with the estimator of the variance, $\widehat{\sigma }$. Higher moments were equated with estimators as follows:

$$\widehat{{{\text{skew}}}} = \frac{{M_{3} }}{{\hat{\sigma }^{3/2} }}$$

$$\widehat{\mathrm{kurtosis}}=\frac{{M}_{4}}{{\widehat{\sigma }}^{2}}$$

Accordingly, the MoM was applied to modelling the Gaussian distributions of the pairwise comparison of RMS differences listed in Table 1. In each case, the MoM approach provides good to excellent fits to each Gaussian distribution. See for example Fig. 4, where the Gaussian distribution for comparison of structures 5ACF and 5ACG, and the resulting distribution calculated from the estimators from the MoM are displayed together over a displacement parameter range of 6–21 Å^2^ (Supplementary Information). Table 1 lists examples of the distribution estimators calculated using the MoM on ranges of displacement parameters.

## Results and discussion

**Fig. 5:**
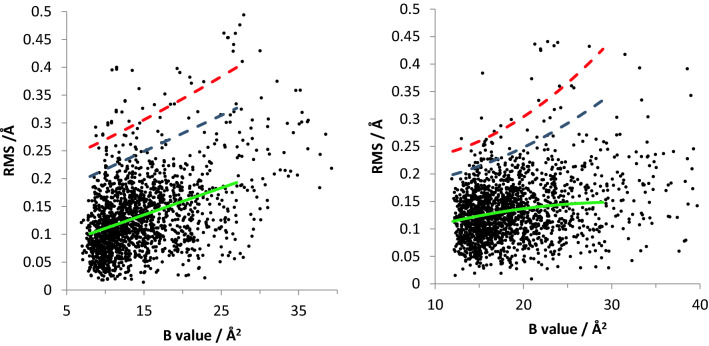
Plots of pairwise differences in atomic positions (left, 5ACF and 5ACG; right 5ACF and 5ACJ), with estimators as a function of *B* value (green = mean, blue = 2 × s.d., red = 3 × s.d.). Note the higher number of significantly shifted atoms in the 5ACF/5ACG comparison (see Discussion)

One significant advantage of using the MoM to provide estimators of statistical parameters is that the use of the equations is computationally trivial, allowing multiple distributions to be analysed. In this regard, to complete the analysis of the distributions of pairwise comparisons of RMS differences between equivalent atoms, we performed the MoM on running ranges of displacement parameters of *x* ± 2 Å^2^, where *x* spans the range of B values for a single pairwise comparison. This analysis was performed for each of the pairwise comparisons listed in Tables 1 and 2. For each pairwise comparison, standard deviations were then calculated for each running range and then used to construct 2 × sd and 3 × sd values as a function of *B* value, which are displayed on the plot of RMS vs displacement parameters for the full data set. An example of such a plot is shown in Fig. 5, which is for the Cu(II) + Cu(II)-G3 (5ACG and 5ACF) comparison. Each point on the plot represents a pairwise comparison of a single equivalent atom between the structures (Supplementary Information Fig. 3). Clusters of atoms associated with a single amino acid side chain, which have RS differences that are greater than the mean + 3 × s.d were taken as significant differences in the position of that amino acid between the two structures.

**Table 2:** Examples of estimators calculated from the MoM of the distributions of RMS differences for the *B* value ranges given in Table 1

Structures	Average ($\widehat{\mu }$)	Standard deviation ($\sqrt{\widehat{\sigma }}$)	Skew	Kurtosis
Cu(I) + Cu(II)	0.11 Å	± 0.056 Å	± 0.018 Å	0.024 Å
5ACH and 5ACG				
Cu(I) + Cu(I)-G3	0.10 Å	± 0.050 Å	± 0.013 Å	0.014 Å
5ACH and 5ACJ				
Cu(II) + Cu(II)-G3	0.13 Å	± 0.059 Å	± 0.014 Å	0.017 Å
5ACG and 5ACF				
Cu(I)-G3 + Cu(II)-G3	0.13 Å	± 0.055 Å	± 0.016 Å	0.022 Å
5ACJ and 5ACF

Each of the pairwise comparisons listed in Tables 1 and 2 is indicative of the structural perturbations brought about by the effects of substrate addition or oxidation state change of the copper ion. In terms of the latter, it is important to note that the differences in Cu(II) and Cu(I) structures are largely due to the fact that reflection data for the former were collected using less intense X-ray exposure to the crystal, which reduces the known photoreduction of the Cu(II) to Cu(I) that is known to occur in X-ray structures of LPMOs [21]. Such conditions also reduce the number and intensity of reflections. Indeed, the quality of the relevant datasets is seen in the pairwise comparisons shown in Table 1 in which those comparisons between Cu(I) structures have lower mean RMS values than those with Cu(II) structures. While the differences in mean RMS values also show the robustness of the current method in assessing errors in comparing the relevant datasets, it is nevertheless important to realise in the analysis below that the Cu(I) structures (5ACH and 5ACJ) derive from X-ray analysis of originally Cu(II) proteins in which photoreduction of the Cu has occurred. Thus the comparison between Cu(I) structures and their Cu(II) counterparts does not represent the structural changes which occur in solution, but those that occur in the solid state at the temperature of the experiment (100 K). With this caveat in mind, those amino acids which show RMS differences greater than 3 × s.d. in their positions between the pairwise structures listed in Tables 1 and 2, are listed in Table 3.

**Table 3:** Lists of amino acids which have significant (> 3 s.d.) differences in the positions of their side chain atoms between the two indicated structures

Structure comparisons	Amino acids significantly shifted between the two structures
Cu(I) + Cu(II)	–
5ACH and 5ACG	
Cu(I) + Cu(I)-G3	Trp5, Glu235
5ACH and 5ACJ	
Cu(II) + Cu(II)-G3	Trp5, Gln162, Tyr203
5ACG and 5ACF	
Cu(I)-G3 + Cu(II)-G3	Glu235
5ACJ and 5ACF	

Glu235 is the C-terminal amino acid and subject to significant positional disorder in the structures.

In the first instance, it can be seen from Table 3 that a simple oxidation state change at the metal ion leads to ostensibly no significant change in the overall structure of the LPMO. Given the caveat raised above, this observation is perhaps of no surprise given that it represents a solid-state change at 100 K brought about by photoreduction. On the other hand, it is notable that the oxidation state change is accompanied by no significant change in the immediate coordination environment of the copper ion (save the coordination of the exogenous ligand), which is commensurate with low reorganisation energy upon electron transfer. Previous cryo-reduction studies of an AA9 LPMO at 77 K which show facile reduction at these temperatures are commensurate with our observations and are indicative of the low barriers to electron transfer which exist in AA9 LPMOs.

**Fig. 6:**
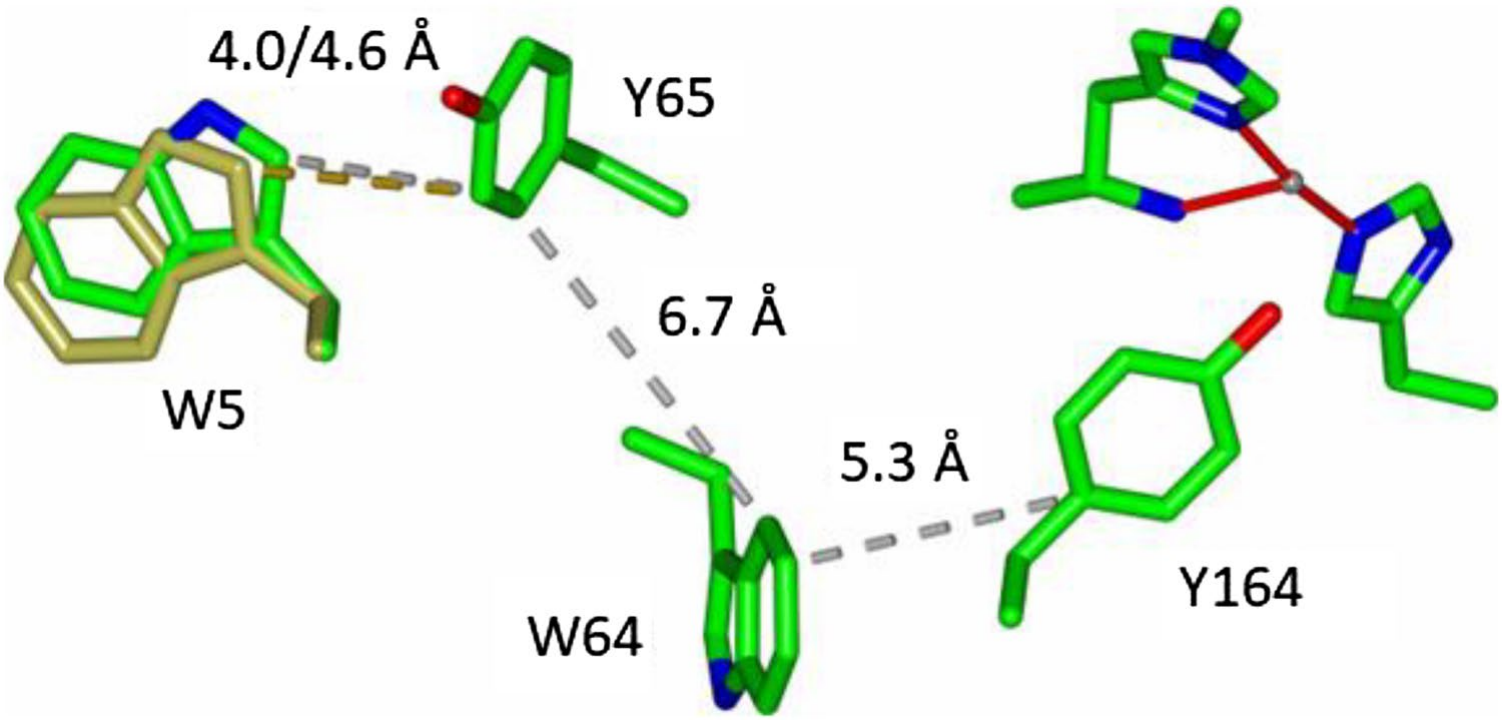
Depictions of the structures of LsAA9 LPMO in the presence and absence of an oligosaccharide substrate (5ACG and 5ACF), showing the amino acid side chains involved in charge transfer to/from the Cu active site, and the change in position of Trp5 brought about by the binding of substrate (W5 represented by gold cylinders shows the position of this residue in the absence of substrate)

The most significant perturbation to the structure of the LPMO is brought about by the addition of the oligosaccharide substrate to both the Cu(I) and Cu(II) forms of the protein. This effect can be seen in Fig. 5 where the number of significantly shifted atoms is clearly greater between the Cu(II) and Cu(II)-G3 structures than the Cu(II) and Cu(I) structures. The shifts occur in the positions of Trp5, Tyr203 and Gln162. Of these shifts, those of Gln162 and Tyr203 are easily correlated with the presence of substrate. Tyr203 lies directly on the substrate-binding face of the LPMO, where Gln162 is an essential active site residue that hydrogen bonds to the exogenous ligand that is coordinated to Cu(II) [22]. Upon binding of substrate this exogenous ligand swaps from OH_2_ to chloride, each of which has a different hydrogen-bonding capacity to Gln162, explaining its shift in position between the two structures. In terms of the shift of Trp5 brought about by the addition of substrate, there is no obvious direct structural link between the substrate and the amino acid side chain that simply explains the difference in the positions of the side chain of Trp5 between the two structures. What is germane to this discussion, however, is that Trp5 has previously been shown to be part of the charge transfer pathway that exists between the active site of the LPMO and the exterior of the protein (Fig. 6) [14].

The likelihood of the shift in the position of Trp5 affecting the rate of charge transfer along the chain depicted in Fig. 5 is small since the rate-limiting step is between Tyr65 and Trp64. However, the position of Trp5 may well be important in charge transfer to an external redox partner that would be necessary to complete the charge transfer pathway. As such, the coupling of substrate binding to the position of the side chain of Trp5 may be important in the ability of the enzyme to transfer charge (e.g. electrons) between external redox partners (e.g. reducing agents) as a function of substrate binding. Such a coupling mechanism would be commensurate with the emerging discussion about the mechanism of substrate activation of the catalytic cycle of LPMOs [12, 23].

## Conclusions

We have presented herein a new means of assessing the random error associated with the comparison of two closely related protein structures. The approach employs the methods of moments (MoM) to derive statistical estimators of RMS differences between atomic positions as a function of their thermal displacement parameters. The advantage of the use of the MoM approach is that accurate and consistent estimators can be easily and quickly calculated by the non-expert for multiple distributions across a range of atomic positions, taking into account any differences in displacement parameter values. As such, the significance of any difference in amino acid positions between structures can be determined, regardless of the thermal parameters associated with the atoms in question. Using this approach, we have analysed the structural changes associated with photoreduction in (100 K) and substrate binding to (room temperature) an AA9 LPMO. Our analysis reveals that there is little structural change upon photoreduction, commensurate with high rates of electron transfer. It also shows that substrate binding is correlated with a small but significant shift in the position of Trp5, and amino acid side chain known to be part of the charge transfer pathway between the active site and exterior of this LPMO. As such, the binding of a substrate may be coupled to the ability of LPMOs to transfer charge to/from the active site during catalysis.

A new paper on the analysis of structural differences of the same AA9 LPMOs described in this paper [24] appeared during a review of this manuscript. Amongst its main conclusions, the authors of this study used global estimates of the error to assess the significance of an ostensible shortening of the Cu…Tyr164 distance upon binding of substrate. Our own analysis, using the methods described herein, on the same Cu…Tyr164 distance suggests that the apparent difference of 0.1–0.2 A between substrate-bound and substrate-free structures is actually within error for both Cu(I) and Cu(II) oxidation states and probably should not be considered as statistically significant.

## Supplementary Information

Below is the link to the electronic supplementary material.

Supplementary file1 (PDF 771 KB)
