# Supplementary material for: Structural perturbations of substrate binding and oxidation state changes in a lytic polysaccharide monooxygenase
Source: J Biol Inorg Chem. 2022 Oct 8;27(8):705–13. doi: 10.1007/s00775-022-01966-z (PMC9653361; doi:10.1007/s00775-022-01966-z)

Supplementary Information for

Structural perturbations of substrate binding and oxidation state changes in a lytic polysaccharide monooxygenase

Paul H Walton* and Gideon J Davies

Department of Chemistry, University of York, Heslington, York, UK, YO10 5DD

Figure 1. Distributions of RMS values (all data). X-axis is pairwise RMS difference between equivalent atoms

Cu(I) – Cu(II)

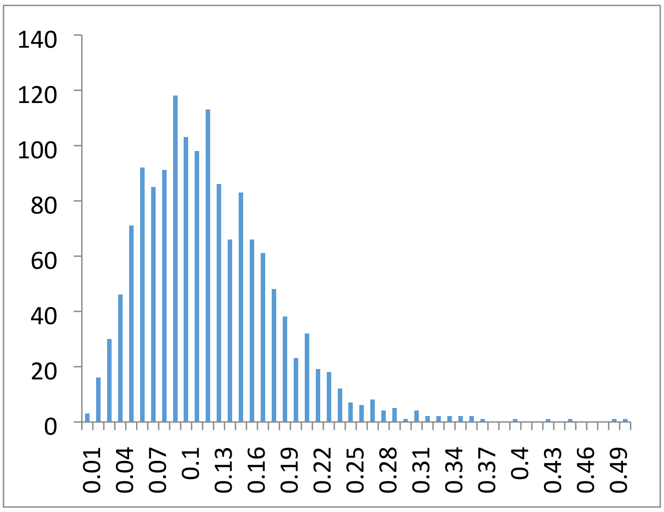

Cu(I) – Cu(I)G3

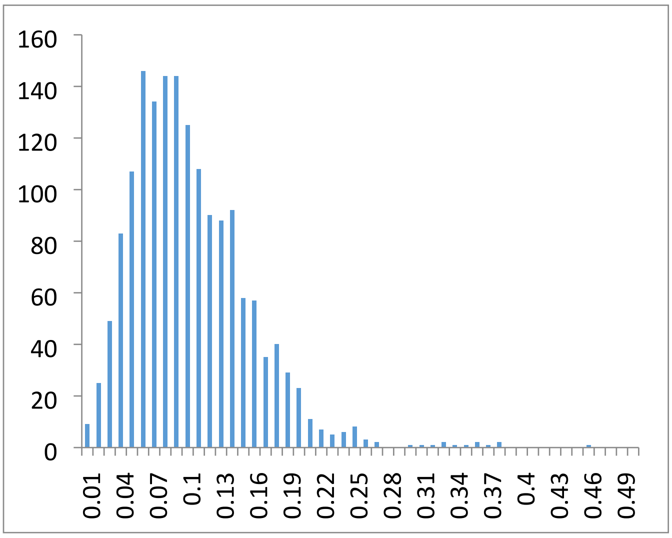

Cu(II) – Cu(II)G3

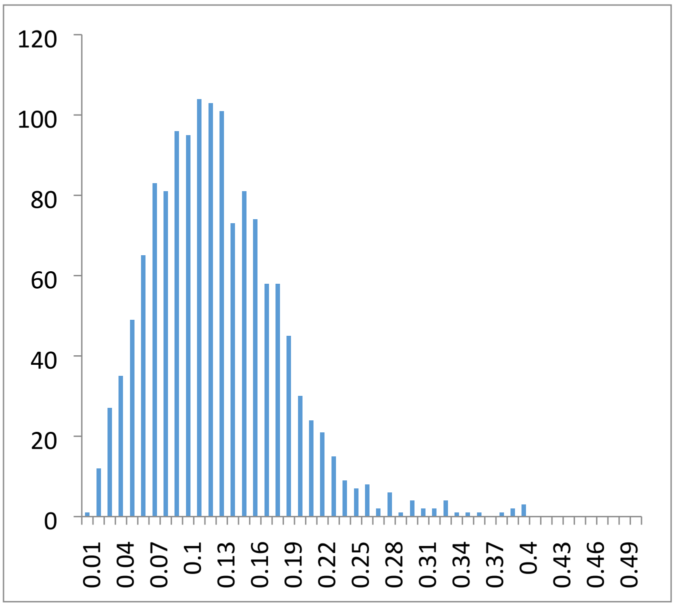

Cu(I)G3 – Cu(II)G3

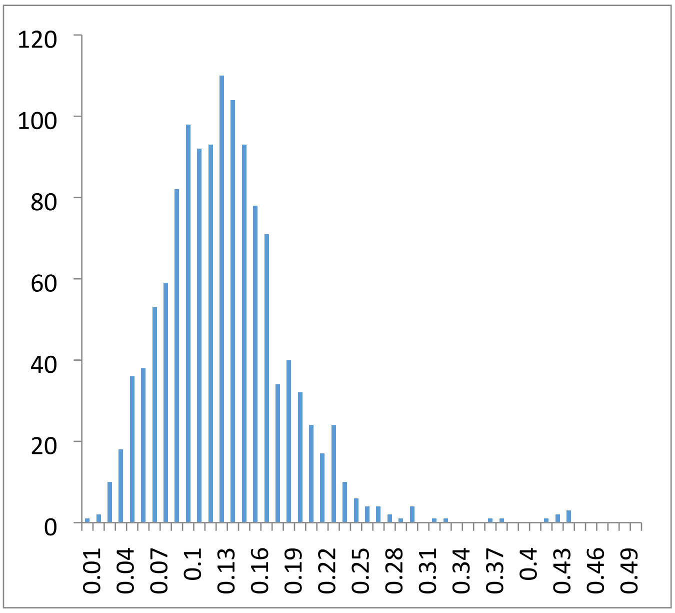

Figure 2. Comparisons of normal distribution histogram (orange columns) calculated from the use MoM parameters in the standard distribution equation for the pairwise comparisons of structures over the displacement parameter ranges as listed in Table 1 of the main text, and experimental data (blue columns). X-axis is pairwise RMS difference between equivalent atoms

Cu(I) – Cu(II)

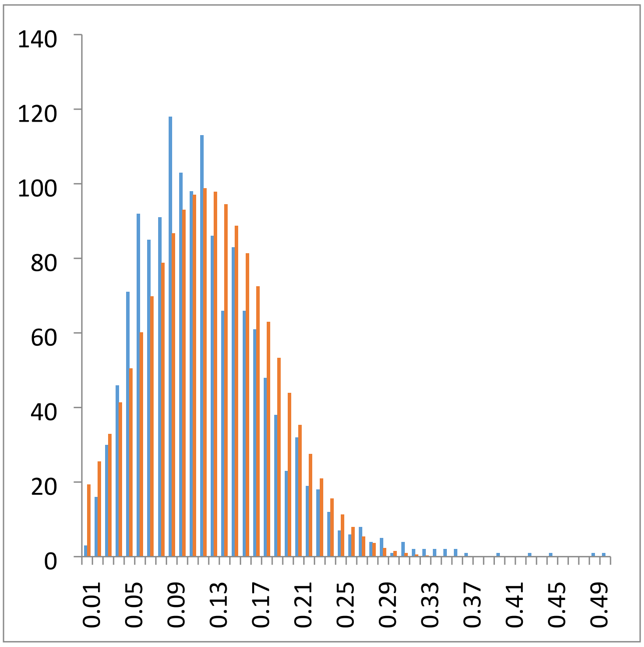

Cu(I) – Cu(I)G3

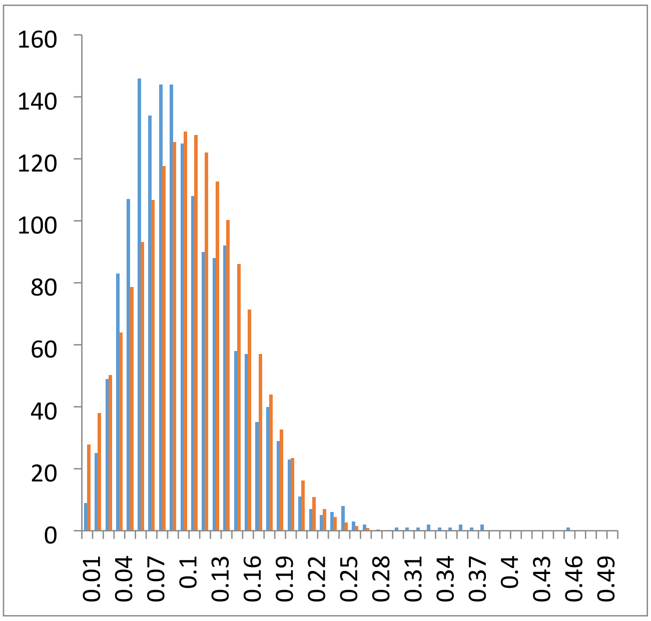

Cu(II) – Cu(II)G3

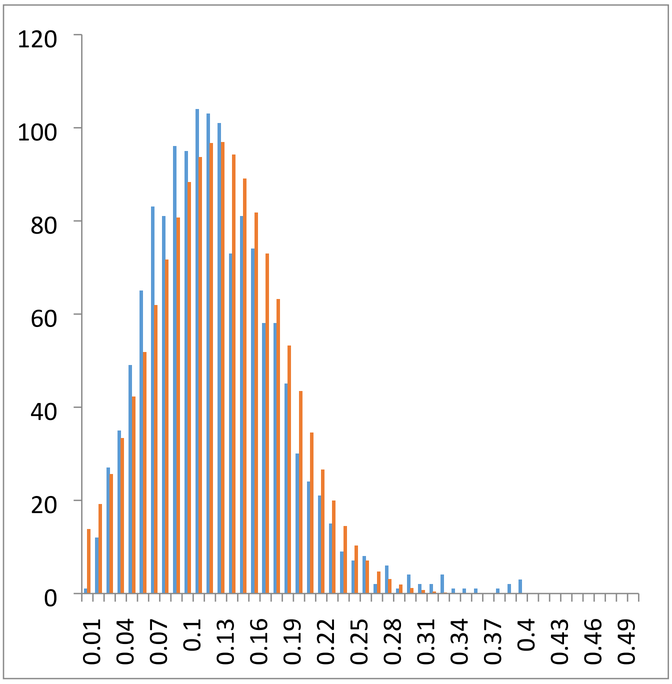

Cu(I)G3 – Cu(II)G3

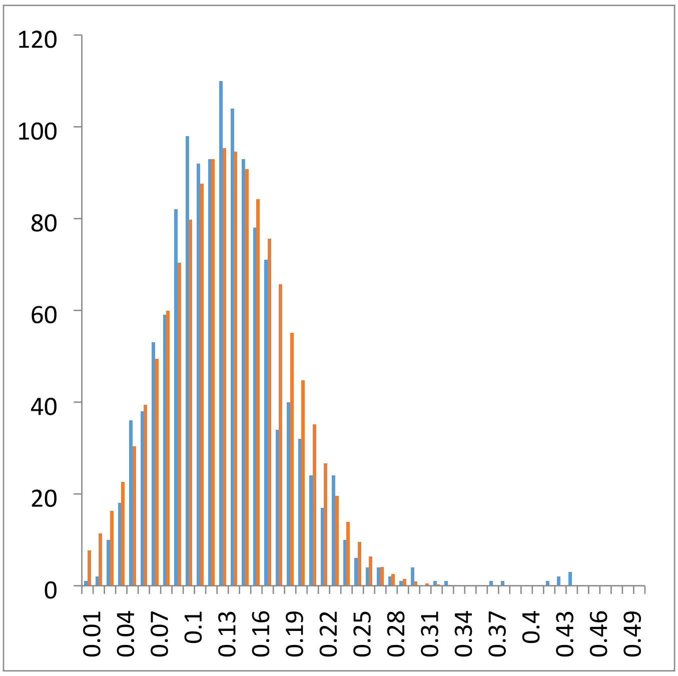

Figure 3. Plots of RMS differences between the atomic positions of pairwise atoms as a function of B-value, with estimators as a function of B value (green =mean, blue = 2 × s.d., red = 3 × s.d.). Vertical dashed lines represent the B-value ranges given in Table 1 of the main text.

Cu(I) – Cu(II)

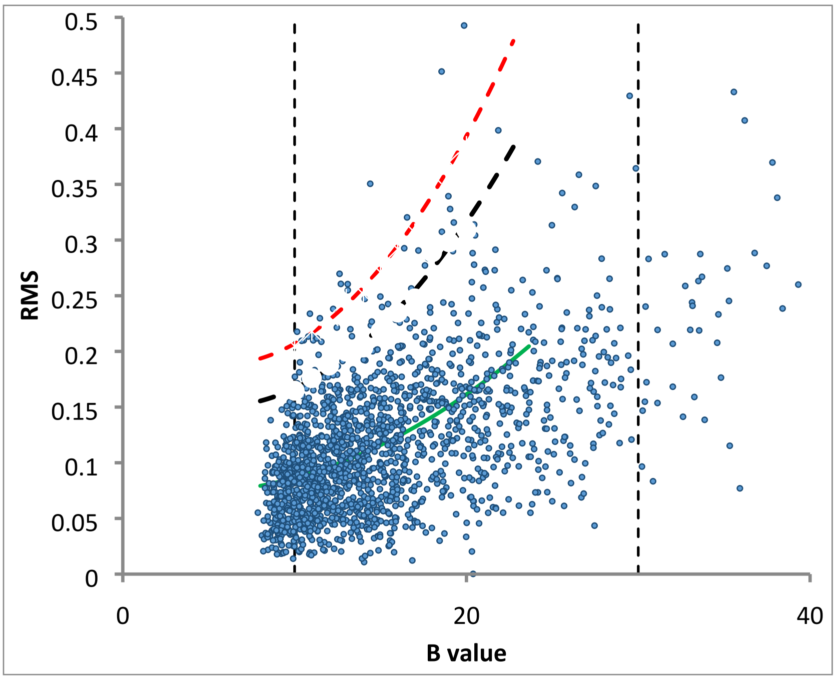

Cu(I) – Cu(I)G3

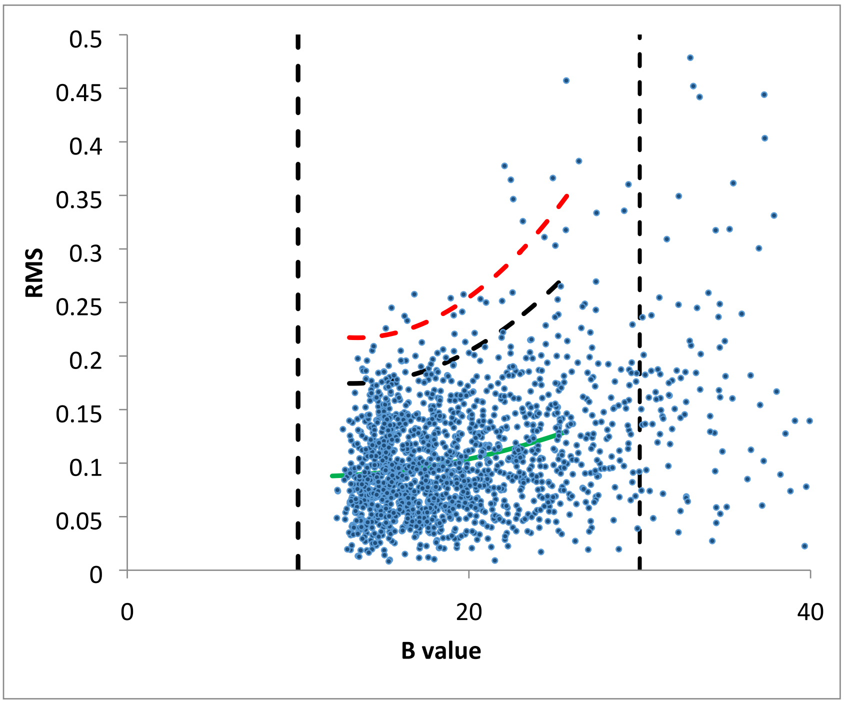

Cu(II) – Cu(II)G3

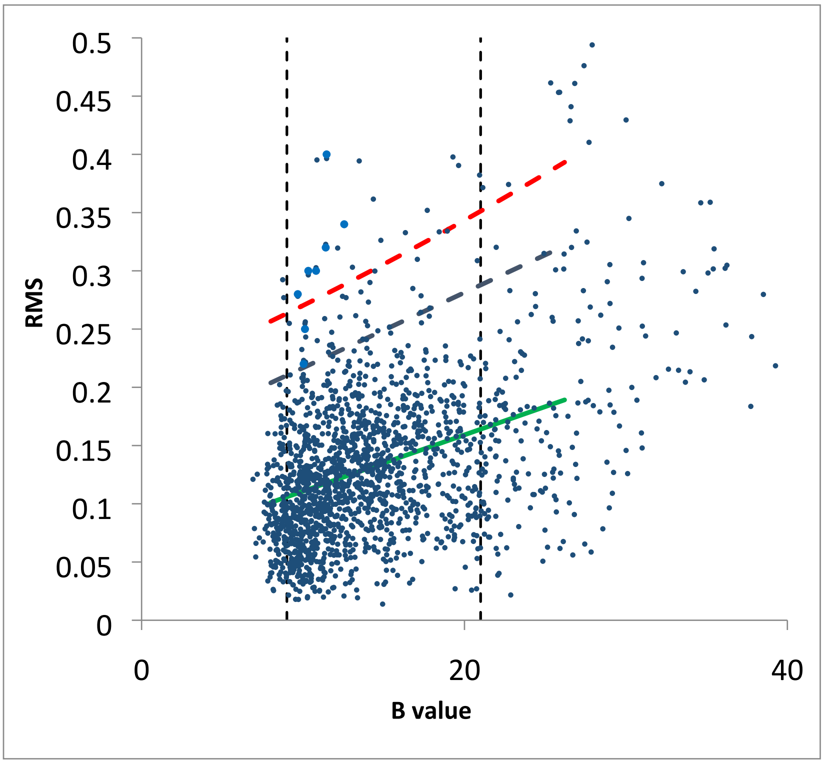

Cu(I)G3 – Cu(II)G3

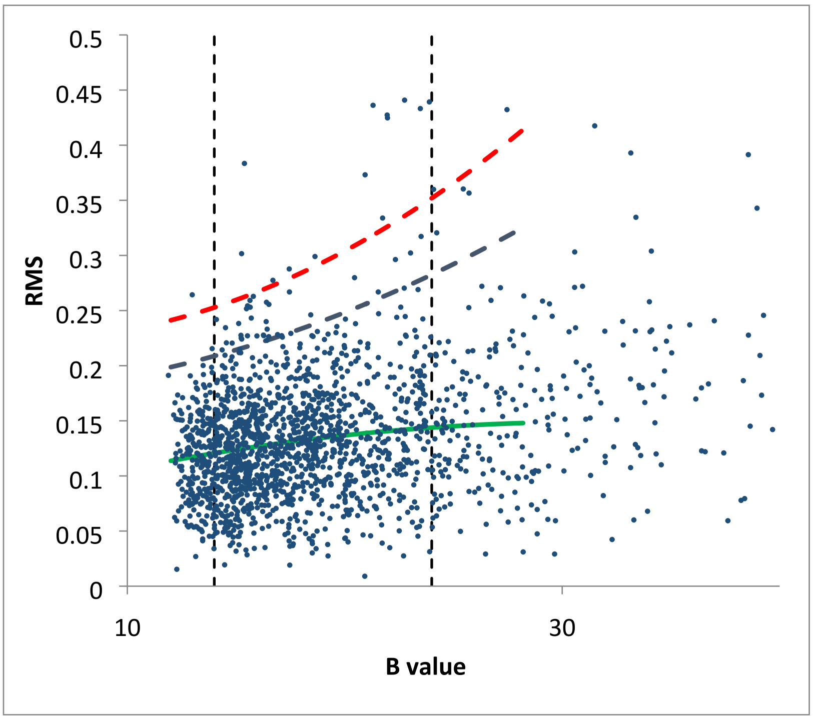

Supplement: Supplementary file 1 — Supplementary file1 (PDF 771 KB) [file 775_2022_1966_MOESM1_ESM.pdf]
